# Comprehension of Complex Sentences in the Persian-Speaking Patients With Aphasia

**DOI:** 10.32598/bcn.9.10.185

**Published:** 2019-05-01

**Authors:** Amir Shiani, Mohammad Taghi Joghataei, Hassan Ashayeri, Mohammad Kamali, Mohammad Reza Razavi, Fariba Yadegari

**Affiliations:** 1. Department of Radiology, Faculty of Allied Medical Sciences, Kermanshah University of Medical Sciences, Kermanshah, Iran.; 2. Cellular and Molecular Research Center, Department of Anatomy, School of Medicine, Iran University of Medical Sciences, Tehran, Iran.; 3. Department of Psychiatry, School of Medicine, Iran University of Medical Sciences, Tehran, Iran.; 4. Department of Basic Rehabilitation Sciences, School of Rehabilitation Sciences, Iran University of Medical Sciences, Tehran, Iran.; 5. Academy of Persian Language and Literature, Tehran, Iran.; 6. Department of Speech Therapy, University of Social Welfare and Rehabilitation Sciences, Tehran, Iran.

**Keywords:** Sentence comprehension, Complex sentences, Syntax, Aphasia

## Abstract

**Introduction:**

To study sentence comprehension in Persian-speaking Patients with Aphasia considering the factors of complexity.

**Methods:**

In this cross-sectional study, the performance of 6 non-fluent aphasic patients were tested and their performance was compared to 15 matched control group. Comprehension of semantically reversible sentences was assessed using a binary sentence-picture matching task. The stimuli were as follows: clefts; subject clefts and object clefts, also relative clauses; subject relatives and object relatives. All of them were types of movement-derived structures and also simple declarative sentences as the control task.

**Results:**

The best performance of aphasic patients were seen in the comprehension of subject clefts, although prior to this result we assumed that simple declarative sentences (in which there is no structural factor of complexity) can be understood easily. They showed the highest difficulty in the comprehension of object relatives. Furthermore, the performance of patients in the comprehension of relative clauses was significantly weaker than understanding the clefts.

**Conclusion:**

The outcomes of this study suggest that the sentence comprehension deficits of aphasic patients, in contrast to the specific deficit models, may not be related to linguistic disabilities. Moreover, the problems in the comprehension of non-canonical sentences may be related to failure in the allocation of attention. Finally, our results support the claims that neural characterization of the cognitive resources (e.g. working memory) is disrupted in sentence comprehension deficits.

## Highlights

- Aphasia refers to the inability to comprehend or produce sentences because of damage to specific brain regions.
- We studied the Persian-speaking patients with aphasia concerning their ability in comprehending different complex sentences.
- Based on the study results, the aphasic patients have the highest ability in the comprehension of subject clefts and the highest difficulty in the comprehension of object relatives.
- The outcomes of this study suggest that the sentence comprehension deficits of aphasic patients may not be related to linguistic disabilities.
- The problems in the comprehension of non-canonical sentences may be caused by difficulty in concentration or cognitive problems such as low working memory in these patients.

## Plain Language Summary

Studies on aphasia are currently conducted on sentence comprehension as well as language production. According to many research studies, patients with aphasia can understand simple sentences but not complex ones. We intended to study comprehension ability of Persian-speaking patients with aphasia concerning different complex sentences. These sentences included subject clefts, object clefts, subject relatives, and object relatives. All of them were types of movement-derived structures. Also, simple declarative sentences were used as the control task. The comprehension of the sentences was assessed using a sentence-picture matching task. Based on the study results, the aphasic patients have the highest ability in the comprehension of subject clefts, although before this result we assumed that simple declarative sentences (in which there is no structural factor of complexity) could be understood easily. They showed the highest difficulty in the comprehension of object relatives. The outcomes of this study suggest that the sentence comprehension deficits of aphasic patients may not be related to linguistic disabilities. Moreover, the problems in the comprehension of non-canonical sentences may be caused by difficulty in concentration. Finally, our results support the claims that cognitive problems such as low working memory is responsible for sentence comprehension deficits in these patients.

## 1. Introduction

Recently, studies on aphasia are focused on sentence comprehension as well as language production (Goodglass & Berko, 1960; Damasio, 1992; Goodglass, 1993). In this regard, the term “syntactic deficits” was proposed to describe the language comprehension deficiencies in aphasic patients at the level of phrases or sentences (Caramazza & Zurif, 1976). Many research studies indicate that Patients With Aphasia (PWA) can understand simple sentences while they have difficulty in the comprehension of complex sentences (Caramazza & Zurif, 1976; Caplan & Futter, 1986; Grodzinsky, 1989; Kim & Thompson, 2000, 2004; Friedmann, Reznick, Dolinski-Nuger, & Soboleva, 2010). The way how Persian aphasics understand complex sentences has not been investigated so far.

There are three interpretations for the inability of sentence comprehension in aphasia. The first is based on cognitive origin and suggests a reduction in the resources (e.g. working memory) that may weaken memory needed for sentence processing (Linebarger, Schwartz & Saffran, 1983; Kolk & van Grunsven, 1985; Caplan, Baker, & Dehaut, 1985; Caplan & Futter, 1986; Naeser et al., 1987; Caplan, Waplan, & Hildebrandt, 1997).

The second, that is called “specific deficit models”, proposes a linguistic origin and postulates that impairment in knowledge is related to specific sentential elements or the inability of using that knowledge in understanding (Linebarger et al., 1983; Mauner, 1995; Beretta, 2001). The third view states that syntactic processing does not recruit one specific area. According to structural and functional neuroimaging evidence, a network of areas, including Broca’s area and anterior, middle, superior, and posterior temporal cortex and adjacent regions are involved in sentence comprehension. Thus, a disruption in the neural network within these areas results in sentence comprehension deficits (Edit & Tamara, 2002; Mack, Meltzer-Asscher, Barbieri, & Thompson 2013; Wilson et al., 2016).

One feature that can make a sentence more difficult to understand is the number of words in it. The comprehension of longer sentences such as relatives -that have embedded clauses- requires more capacity to maintain representation in memory. However, PWAs are pathologically deprived of these resources, i.e. working memory (Caplan & Waters, 1999).

It should be noted that sentence structure also plays an important role in determining how challenging particular sentences are to understand. In this context, one factor of structural complexity relates to the syntactic movement (Caramazza & Zurif, 1976). Many researchers maintain when an element of one sentence moves from its original position to another position, PWAs have problems to assign a semantic role to the moved element. Studies of comprehension of syntactic complexity in various languages have found that PWAs show the worst difficulties in the comprehension of sentences with the moved structures and non-canonical word order (Caramazza & Zurif, 1976; Friedmann et al., 2010).

These findings mentioned the order of the words in a sentence as the important signal of syntactic complexity. The canonical word order is the most usual order of main sentential elements (Menn, 2000; Jacobs & Thompson, 2000). Although, in English and some other languages to make a canonical word order the verb comes between agent and theme (Caramazza & Zurif, 1976; Heilman & Scholes, 1976; Schwartz, Saffran, & Marin, 1980; Saffran, Berndt, Schwartz, 1989), in the Persian language the usual order of sentential elements is agent -theme -verb (Brunner 1977; Dabir-Moghaddam, 2001; Dabir-Moghaddam, 2006).

Sentences with non-canonical word order such as object relatives and object clefts are more complex than those with canonical word order such as subject relatives and subject clefts (Kiran et al., 2012). So, one feature of our study is to determine the hierarchical level of complexity from less complex to more complex structures as it has not been investigated in the Persian aphasics yet. This issue becomes more important in recent studies, as one of the common therapeutic approaches is based on the Complexity Account of Treatment Efficacy (CATE) (Thompson & Shapiro, 2007). CATE states that the training on more complex structures leads to generalization to less complex structures.

Research indicates that in the presence of syntactic factors of complexity in a sentence, PWAs are able to use their lexical-semantic knowledge as a cue to comprehend that sentence. In this regard, PWAs may understand a non-canonical sentence with irreversible semantic roles, since its semantic relations could be used as a cue (Sherman & Schweickert 1989; Friedmann et al., 2010). For this reason, in the current study, the semantic roles of all sentences were reversible. It is also interesting to explore whether the comprehension problems of non-canonical sentences are due to lack of attention or loss of knowledge about certain syntactic constituents. Finally, as a network of a brain area is involved in sentence comprehension and not one specific area, so we examined the comprehension of complex sentences in all non-fluent aphasic patients, including patients with Broca and agrammatic aphasia.

## 2. Methods

### 2.1. Study participants

The study participants were 6 aphasic patients and 15 individuals without communicative impairments. All participants were monolingual Persian-speakers, aged from 40 to 70 years (Mean±SD age: 57±7.8 y). They were divided into three subgroups: 40–50, 50–60, and 60–70 years old. All of the participants were right-handed. They had at least 12 years of formal education, with no history of neurodegenerative diseases or apraxia. The aphasic patients were stroke survivors with a single lesion in the left anterior cerebral hemisphere. They were in the chronic phase of recovery and 8 to 30 months (Mean±SD duration: 20±6.5 mon) have passed their strokes.

The patients’ scores on the Persian Aphasia Test (Nilipour, Pour Shahbaz, Ghoreishi, Yousefi, 2016) indicated their non-fluent aphasic problems and also their speech was characterized by limited production of sentences, especially complex structures. They had received different periods of language therapy before participating in this study, but their therapeutic programs did not specifically focus on their problems in complex sentences. The patients with difficulties in the comprehension of nouns and verbs used in the tasks of this study were excluded from sampling. The matched-control participants were 15 Persian-speaker individuals without language disorder. Five control participants were age-matched to each subgroup of the patients.

### 2.2. Study task

**Figure 1.**
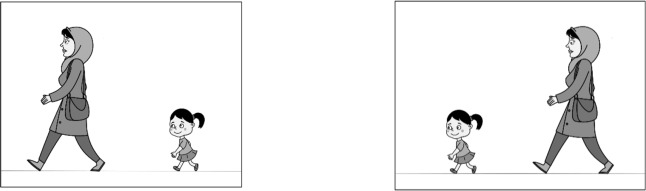
A sample of a picture pair used in the binary sentence-picture matching tasks

The task of the current study comprised 70 reversible sentences. Ten simple declarative sentences (with no structural factor of complexity) and 60 movement-driven sentences consisting of 30 clefts: 15 subject clefts and 15 object clefts; and 30 relative clauses: 15 subject relatives and 15 object relatives. Both the object clefts and object relatives had non-canonical structures. A transitive action verb was used for all sentences, these verbs were divalent so needed an agent and a theme as semantic roles. The semantic roles of the sentences were reversible (Figure 1). All noun phrases were singular (Table 1). The sentences were constructed by the researcher and all matching pictures were drawn by the same artist. The presentation of sentences was in random order.

**Table 1.** Samples of target sentences in the task

**Type**	**Semantic Reversible Sentences**
Simple declarative	/mâdar/ /bače/ /ra/ /donbal/ /mikonad/. The mother is following the child.
Subject cleft	/in/ /bače/ /Ɂst/ /ke/ /mâdar/ /ra/ /donbal/ /mikonad/. This is the child who is following the mother.
Object cleft	/in/ /mâdar/ /Ɂst/ /ke/ /bače/ /Ɂu/ /ra/ /donbal/ /mikonad/. This is the mother whom the child is following.
Subject relative	/mâdari/ /ke/ /bače/ /ra/ /donbâl/ /mikonad/ /be/ /ĵelo/ /negâh/ /mikonad/. The mother who is following the child is looking forward.
Object relative	/mâdari/ /ke/ /bače/ /donbâlash/ /mikonad/ /be/ /ĵelo/ /negâh/ /mikonad/. The mother whom the child is following is looking forward.

### 2.3. Study procedure

A double choice sentence to picture matching task was used to assess the performance of participants regarding the sentence comprehension. In these tasks, two pictures were presented for each sentence; a target picture which showed the event in the sentence and a foil picture in which the semantic roles were reversed. The frequency of the position of the target picture on either side of the screen was counterbalanced during the testing in each trial.

The written sentence was read aloud by the examiner and the participants were asked to choose the correct picture. The examiner would read each sentence only once more if the participant requested. The task was presented without time limit.

### 2.4. Reliability

We examined the inter-observer reliability of responses. Thus, all responses of each subject were scored by the primary examiner and a trained, independent judge. Point-to-point agreement ranged from 90% to 100% (Mean=95%).

### 2.5. Data analysis

The data were analyzed at the group level since both patient and control participants were homogeneous. As the data showed the normal distribution, we used parametric tests. The performance of the patients was compared with the performance of the control group by the Independent t-test. The performance of the three subgroups in each aphasic and control participants were compared by 1-way ANOVA, regarding each sentence structure. To examine the effect of structure regarding sentence comprehension in the patient group, we compared the performance in every two related structures using the paired t-test.

## 3. Results

### 3.1. Control participants

The performance of the control group was at the ceiling level for all sentence types, and their percentages of correct responses were between 93% and 100% during all types of sentences. Every three subgroups was matched with a different subgroup of the patient, differences between subgroups were analyzed using 1-way ANOVA, regarding each sentence structure. There was no significant difference between subgroups.

### 3.2. Control and patient groups

The results of comparisons between patient and control participants were done by the Independent samples t-test which showed that in all structures and especially in more complex types, the performance of patients were significantly weaker than the controls (P<0.01).

### 3.3. Patient group

The mean percentage of accuracy and standard deviation of patients’ performance in all sentence types of this study are shown in Tables 2 separately. Also, the position of sentences in different levels of complexity can be seen in the Tables 2 and 3. As seen in Tables 2, only the performance of patients in subject clefts (74%) and simple declarative sentence (71%) task was above the chance level, also the best comprehension of sentences was allocated to the subject clefts (74%) task.

**Table 2.** The performance of all patients with respect to each type of sentence (Mean±SD number and percentage of correct responses) (n=6)

**Variables**	**Semantic Reversible**				

	**Movement-Derived**				**Simple Declarative Sentences**

	**Non-Canonical**		**Canonical**		

	**Object Relatives**	**Object Clefts**	**Subject Relatives**	**Subject Clefts**	
Mean	23% (4 out of 15)	33% (5 out of 15)	40% (6 out of 15)	7% (11 out of 15)	71% (7 out of 10)
SD	0.0687	0.0526	0.7731	0.0526	0.0752

**Table 3.** Comparison of relatives and clefts sentences (Mean±SD number and percentage of correct responses) (n=6)

**Variables**	**Semantic Reversible**	

	**Relatives**	**Clefts**
Mean	31% (9 out of 30)	53% (16 out of 30)
SD	0.0658	0.0516
P	<0.001	

On the other hand, their performance in the comprehension of subject relatives (40%) task was a little below the chance level and they were also significantly weak in the comprehension of object clefts (33%) and object relatives (23%) tasks, too. Therefore we considered non-canonical sentences as more complex structures, although the patients showed the lowest performance in the comprehension of the object relatives. Consequently, the pairwise comparison between simple declarative sentences and movement-derived structures, canonical versus non-canonical structures, and clefts versus relative clauses was done by the paired t-test.

#### 3.3.1. Clefts vs. relative clauses

To find out the level of complexity between clefts and relative clauses, the average performance of patients’ comprehension in these tasks was compared. As it is shown, each of these parts consisted of clefts (subject cleft and object clefts) and relatives (subject relatives and object relatives) structures. Results showed that the patients performed at the chance level in the comprehension of clefts (53%) but their performance in relative clauses was below the chance level (31%). Therefore the comprehension of relative clauses was significantly lower than the comprehension of clefts (P=0.000) (Table 3).

#### 3.3.2. Simple declarative vs. movement-derived structures:

The movement in the constituent of the sentence from its base is a factor of complexity. The average performance of patients in movement-derived sentences task was compared with their performance in simple declarative sentence task. As mentioned before, movement-derived sentences included all clefts and relative clauses. Although the average performance of patients in movement-derived sentences was at the chance level, the patient group showed a significant difference in the comprehension of these two types of structures (P<0.000) (Table 4).

**Table 4.** Comparison of simple declarative and movement-derived structures (Mean±SD percentage and number of correct responses) (n=6)

**Variables**	**Semantic Reversible**	

	**Movement-Derived**	**Simple Declarative**
Mean	48% (29 out of 60)	71% (7 out of 10)
SD	0.236	0.0752
P	<0.001	

#### 3.3.3. Canonical vs. non-canonical structures

To investigate the effect of word order in sentences, the performance of patients in the comprehension of canonical (subject relatives and subject clefts) and non-canonical sentences (object relatives and object clefts) were compared. As presented in Table 5, the patients group performed below the chance level in the comprehension of non-canonical sentences (28%) and as noted above, comparisons of pair structures in the patient group were done by the paired t-test. It means that they performed significantly better in the comprehension of canonical sentences (P=0.000).

**Table 5.** Comparison of canonical and non-canonical structures (Mean±SD percentage and number of correct responses) (n=6)

**Variables**	**Semantic Reversible**	

	**Non-Canonical**	**Canonical**
Mean	28% (8 out of 30)	57.4% (17 out of 30)
SD	0.0806	0.0539
P	<0.0001	

**Figure 2.**
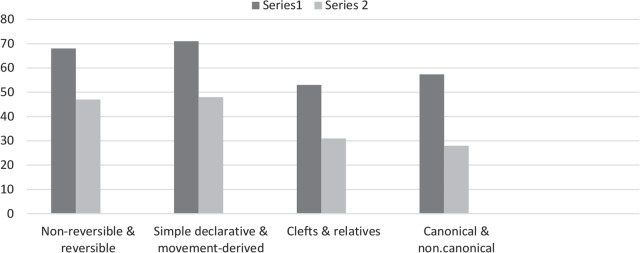
Comprehension of each paired structures in the patient group (percentage of correct responses)

To sum up, the average performance of patients in all three conditions of complexity versus simple matched pairs are shown below in Figure 2 (Series 2 shows comprehension in the condition of complexity: movement-derived, non-canonical and relative, and the series 1 shows the performance in their matched simple conditions: simple declarative, canonical and cleft, respectively).

## 4. Discussion

This study investigated the comprehension of sentences considering the factors of complexity in Persian-speaking PWAs. The major goals were to restrict the study to syntactic aspects by using semantically reversible sentences and to determine the level of complexity in related structures for using them in the clinical setting.

According to the obtained results, the matched control group performed normally in all sentence types, and their performance was significantly different from the patients’ performance. Furthermore, neither normal nor patient participants had significant differences between their three age-ranged subgroups. In contrast, some studies have found that elderly subjects generally perform worse in understanding complex sentences (Feier & Gerstman, 1980). Other studies have found lower comprehension of more complex syntactic structures in the elderly (Obler, Fein, Nicholas & Albert, 1991; Davis & Ball, 1989). This inconsistency may be attributed to the higher educational level of the participants of this study.

On the other hand, PWAs showed problems in understanding of complex sentences, and their performance in pair structures would be described as follows. Similar to English-speaking PWAs, the Persian-speaking ones showed significantly lower accuracy in the comprehension of relative clauses compared to the comprehension of the clefts (Friedmann et al., 2010; Duman, Altınok, Özgirgin, & Bastiaanse, 2011; Kiran et al., 2012).

Both of these structures were similar in syntactic complexity. In addition, the semantic roles were reversible for both types. So, the only difference between them was the sentence length that is shorter in clefts. Clearly, there is a negative correlation between the length of sentence and accuracy of performance in a way that the longer the sentence, the lower the performance was (Cooke et al., 2002). The comprehension of longer sentences requires more capacity to maintain representation in memory. However, PWAs are pathologically deprived of these resources (i.e. working memory) (Caplan, Waters, & Michaud, 2011, Reali & Christiansen, 2007; Naeser et al., 1987; Caplan et al., 1997).

The present study also compared the performance of PWAs in the comprehension of canonical sentences (i.e. subject clefts and subject relatives) versus non-canonical sentences (i.e. object clefts and object relatives). PWAs performed significantly below the chance level in the comprehension of non-canonical sentences. This finding is consistent with previous studies on aphasic sentence comprehension in some other languages that showed PWA’s comprehension is especially impaired in non-canonical sentences, with no difficulty in canonical sentences (Friedmann et al., 2010).

The canonical word order is the most frequently-used structure in any language, in comparison with non-canonical sentences which are rare. Thus, most individuals may judge non-canonical structures based on the ordinary form of sentence which needs more attention to distinguish the unusual form of the sentence (Salthouse, 1996). Therefore, PWAs “tend to fall back on the dominant word order in judging agent-theme relations” (Dick et al., 2001). In addition, PWAs have limitations in the allocation of attention resources to these infrequent non-canonical sentences (Haarmann, Just, & Carpenter 1997).

The results showed that PWAs’ performance was at a chance level compared to movement-derived sentences. On the other hand, although the comprehension of simple declarative was not at the highest level, it was significantly above the chance level. The movement-derived sentences in this study consisted of both canonical and non-canonical structures. For this reason, the low performance of PWAs is related to the understanding of the non-canonical sentences, with no relation to the features of movement elements. In addition, they performed above the chance level compared to subject clefts as a type of movement-derived structure; this performance was even better than their performance in the comprehension of simple declarative sentences, in which there is no factor of syntactic complexity (Table 2). Therefore, these results are inconsistent with the theory suggesting that movement of noun phrases is impaired in PWAs (Trace Deletion Hypotheses, Grodzinsky, 1989, 2000).

Syntactic complexity that would lead to comprehension deficits even in the shorter sentences (i.e. object clefts) is related to the problems in the allocation of attention. Additionally, working memory problem is the main source of a misunderstanding of longer sentences (i.e. relatives). In other words, there is a direct relationship between sentence length and working memory. These clinical observations argue against hypotheses that posit a deficit of a specific linguistic function and favors the evidence from neuroimaging that sentence comprehension deficits is due to disruption in neural connections within the relevant brain areas (Mack, et al., 2013; Wilson et al., 2016).

This study has also implications that might be used in the diagnosis and treatment of aphasia, such as determining the level of complexity between the relevant structures in the Persian language (i.e. relatives>clefts, object relatives>subject relatives, object clefts>subject clefts, and simple declarative sentences>subject clefts). These hierarchical level could be useful in planning treatment in aphasia therapy (Thompson & Shapiro, 2007; Thompson et al., 2003).

## References

[B1] Beretta A. (2001). Linear and structural accounts of theta-role assignment in agrammatic aphasia. Journal of Aphasiology, 15(6), 515–31. [DOI:10.1080/02687040143000023]

[B2] Brunner Ch. (1977). A Syntax of Western Middle Iranian Caravan Books. New York: Delmar.

[B3] Caplan D. Futter C. (1986). Assignment of thematic roles to nouns in sentence comprehension by an agrammatic patient. Journal of Brain and Language, 27(1), 117–34. [DOI:10.1016/0093-934X(86)90008-8] 3947937

[B4] Caplan D. Waters G. S. (1999). Verbal working memory and sentence comprehension. Behavioral and Brain Sciences, 22(1), 77–94. [DOI:10.1017/S0140525X99001788] [PMID ] 11301522

[B5] Caplan D. Baker C. Dehaut F. (1985). Syntactic determinants of sentence comprehension in aphasia. Cognition, 21(2), 117–75. [DOI:10.1016/0010-0277(85)90048-4] 2419022

[B6] Caplan D. Waters G. S. Hildebrandt N. (1997). Determinants of sentence comprehension in aphasic patients in sentence-picture matching. Journal of Speech, Language and Hearing Research, 40(3), 542–55. [DOI:10.1044/jslhr.4003.542] 9210113

[B7] Caplan D. Waters G. DeDe G. Michaud J. (2011). Effects of age, speed of processing and working memory on comprehension of sentences with relative clauses. Journal of Psychology and Aging, 26(2), pp. 439–50. [DOI:10.1037/a0021837] [PMID ] 21480714

[B8] Caramazza A. Zurif E. (1976). Dissociation of algorithmic and heuristic processes in language comprehension: Evidence from aphasia. Journal of Brain and Language, 3(4), 572–82. [DOI:10.1016/0093-934X(76)90048-1] 974731

[B9] Cooke A. Zurif E. B. DeVita C. Alsop D. Koenig P. Detre J. (2002). Neural basis for sentence comprehension: Grammatical and short-term memory components. Journal of Human Brain Mapping, 15(2), 80–94. [DOI:10.1002/hbm.10006] [PMID ] 11835600 PMC6872024

[B10] Dabir-Moghaddam M. (2001). Word order typology of Iranian languages. The International Journal of Humanities, 8(2), 17–23.

[B11] Dabir-Moghaddam M. (2006). Internal and external forces in typology: Evidence from Iranian languages. Journal of Universal Language, 7(1), 29–47. [DOI:10.22425/jul.2006.7.1.29]

[B12] Damasio A. (1992). Aphasia. New England Journal of Medicine, 326(8), 531–9. [DOI:10.1056/NEJM199202203260806] 1732792

[B13] Davis C. A. Ball H. E. (1989). Effects of age on comprehension of complex sentences in adulthood. Journal of Speech and Hearing Research, 32(1), 143–50. [DOI:10.1044/jshr.3201.143] 2704189

[B14] Dick F. Bates E. Wulfeck B. Utman J. A. Dronkers N. Gernsbacher M. A. (2001). Language deficits, localization, and grammar: Evidence for a distributive model of language breakdown in aphasic patients and neurologically intact individuals. Psychological Review, 108(4), 759–88. [DOI:10.1037/0033-295X.108.4.759] [PMID ] [PMCID ] 11699116 PMC4301444

[B15] Edith K. Tamara Y. (2002). The brain circuitry of syntactic comprehension. Trends in Cognitive Sciences, 6(8), 350–6. [DOI:10.1016/S1364-6613(02)01947-2] 12140086

[B16] Feier C. D. Gerstman L. J. (1980). Sentence comprehension abilities throughout the adult life span. Journal of Gerontology, 35(5), 722–8. [DOI:10.1093/geronj/35.5.722] [PMID ] 7430569

[B17] Friedmann N. Shapiro L. (2003). Agrammatic comprehension of simple active sentences with moved constituents: Hebrew OSV and OVS structures. Journal of Speech, Language and Hearing Research, 46(2), 288–97. [DOI:10.1044/1092-4388(2003/023)] PMC3392331 14700372

[B18] Friedmann N. Reznick J. Dolinski-Nuger D. Soboleva K. (2010). Comprehension and production of movement-derived sentences by Russian speakers with agrammatic aphasia. Journal of Neurolinguistics, 23(1), 44–65. [DOI:10.1016/j.jneuroling.2009.08.002]

[B19] Goodglass H. (1993). Understanding aphasia. San Diego, California: Academic Press.

[B20] Goodglass H. Berko J. (1960). Agrammatism and inflectional morphology in English. Journal of Speech and Hearing Research, 3(3), 257–67. [DOI:10.1044/jshr.0303.257] 13851047

[B21] Grodzinsky Y. (1986). Language deficits and syntactic theory. Brain and Language, 27(1), 135–59. [DOI:10.1016/0093-934X(86)90009-X] 3947938

[B22] Grodzinsky Y. (1989). Agrammatic comprehension of relative clauses. Brain and Language. 37(3), 480–99. [DOI:10.1016/0093-934X(89)90031-X] 2478254

[B23] Grodzinsky Y. (2000). The neurology of syntax: Language use without Broca’s area. Behavioral and Brain Sciences, 23(1), 1–71. [DOI:10.1017/S0140525X00002399] [PMID ] 11303337

[B24] Haarmann H. J. Just M. A. Carpenter P. A. (1997). Aphasic sentence comprehension as a resource deficit: A computational approach. Brain and Language, 59(1), 76–120. [DOI:10.1006/brln.1997.1814] [PMID ] 9262852

[B25] Heilman K. Scholes R. (1976). The nature of comprehension errors in Broca’s, conduction, and Wernicke’s aphasics. Cortex, 12(3), 258–65. [DOI:10.1016/S0010-9452(76)80007-X] 1000994

[B26] Jacobs B. J. Thompson C. K. (2000). Cross-modal generalization effects of training noncanonical sentence comprehension and production in agrammatic aphasia. Journal of Speech, Language, and Hearing Research, 43(1), 5–20. [DOI:10.1044/jslhr.4301.05] PMC3025283 10668649

[B27] Kim M. Thompson C. K. (2000). Patterns of comprehension and production of nouns and verbs in agrammatism: Implications for lexical organization. Brain and Language, 74(1), 1–25. [DOI:10.1006/brln.2000.2315] [PMID ] [PMCID ] 10924214 PMC4423608

[B28] Kim M. Thompson C. K. (2004). Verb deficits in Alzheimer’s disease and agrammatism: Implications for lexical organization. Brain and Language, 88(1), 1–20. [DOI:10.1016/S0093-934X(03)00147-0] 14698726 PMC3079403

[B29] Kiran S. Caplan D. Sandberg C. Levy J. Berardino A. Villard S. (2012). Development of a theoretically based treatment for sentence comprehension deficits in aphasia. American Journal of Speech-Language Pathology, 21(2), 88–102. [DOI:10.1044/1058-0360(2012/11-0106)] PMC3348417 22411773

[B30] Kolk H. H. Van Grunsven M. M. (1985). Agrammatism as a variable phenomenon. Cognitive Neuropsychology, 2(4), 347–84. [DOI:10.1080/02643298508252666]

[B31] Linebarger M. C. Schwartz M. F. Saffran E. M. (1983). Sensitivity to grammatical structure in so-called agrammatic aphasics. Cognition, 13(3), 361–92. [DOI:10.1016/0010-0277(83)90015-X] 6683142

[B32] Mack J. Meltzer-Asscher A. Barbieri E. Thompson C. (2013). Neural correlates of processing passive sentences. Brain Sciences, 3(3), 1198–214. [DOI:10.3390/brainsci3031198] 24961525 PMC4061884

[B33] Mauner G. (1995). Examining the empirical and linguistic bases of current theories of agrammatism. Brain and Language, 50(3), 339–68. [DOI:10.1006/brln.1995.1052] [PMID ] 7583194

[B34] Menn L. (2000). It’s time to face a simple question: What makes canonical form simple. Brain and Language, 71(1), 157–9. [DOI:10.1006/brln.1999.2239] 10716834

[B35] Naeser M. A. Mazurski P. Goodglass H. Peraino M. Laughlin S. Leaper W. C. (1987). Auditory syntactic comprehension in nine aphasia groups (with CT scans) and children: Differences in degree but not order of difficulty observed. Cortex. 23(3), 359–80. [DOI:10.1016/S0010-9452(87)80001-1] 3677727

[B36] Nilipour R. Pour Shahbaz A. Ghoreishi Z. S. Yousefi A. (2016). [Reliability and validity of Persian aphasia battery test (Persian)]. Iranian Journal of Ageing, 10(4), 182–91.

[B37] Obler L. K. Fein D. Nicholas M. Albert M. L. (1991). Auditory comprehension and aging: Decline in syntactic processing. Applied psycholinguistics, 12(4), 433–52. [DOI:10.1017/S0142716400005865]

[B38] Reali F. Christiansen M. H. (2007). Processing of relative clauses is made easier by frequency of occurrence. Journal of Memory and Language, 57(1), 1–23. [DOI:10.1016/j.jml.2006.08.014]

[B39] Saffran E. M. Berndt R. S. Schwartz M. F. (1989). The quantitative analysis of agrammatic production: Procedure and data. Brain and Language, 37(3), 440–79. [DOI:10.1016/0093-934X(89)90030-8] 2804622

[B40] Salthouse T. A. (1996). The processing-speed theory of adult age differences in cognition. Psychological Review, 103(3), 403–28. [DOI:10.1037//0033-295X.103.3.403] 8759042

[B41] Schwartz M. F. Saffran E. Marin O. (1980). The word order problem in agrammatism. I: Comprehension. Brain and Language, 10(2), 249–62. [DOI:10.1016/0093-934X(80)90055-3] 7407546

[B42] Sherman J. C. Schweickert J. (1989). Syntactic and semantic contributions to sentence comprehension in agrammatism. Brain and Language, 37(3), 419–39. [DOI:10.1016/0093-934X(89)90029-1] 2478253

[B43] Thompson C. K. Shapiro L. P. (2007). Complexity in Treatment of Syntactic Deficits. American Journal of Speech Language Pathology, 16(1), 30–42. [DOI:10.1044/1058-0360(2007/005)] 17329673 PMC2238729

[B44] Thompson C. K. Shapiro L. P. Kiran S. Sobecks J. (2003). The role of syntactic complexity in treatment of sentence deficit in agrammatic aphasia: The Complexity Account of Treatment Efficacy (CATE). Journal of Speech, Language and Hearing Research, 46(3), 591–607. [DOI:10.1044/1092-4388(2003/047)] PMC1995234 14696988

[B45] Wilson S. M. DeMarco A. T. Henry M. L. Gesierich B. Babiak M. Miller B. L. (2016). Variable disruption of a syntactic processing network in primary progressive aphasia. Brain, 139(11), 2994–3006. [DOI:10.1093/brain/aww218] 27554388 PMC5091045

[B46] Duman T. Y. Altınok T. Özgirgin N. Bastiaanse R. (2011). Sentence comprehension in Turkish Broca’s aphasia: An integration problem. Journal of Aphasiology, 25(8), 908–26. [DOI:10.1080/02687038.2010.550629]

